# Mycochemical Characterization of* Agaricus subrufescens* considering Their Morphological and Physiological Stage of Maturity on the Traceability Process

**DOI:** 10.1155/2017/2713742

**Published:** 2017-09-10

**Authors:** Diego Cunha Zied, Jose E Pardo, Rafael Simões Tomaz, Celso Tadao Miasaki, Arturo Pardo-Giménez

**Affiliations:** ^1^Faculdade de Ciências Agrárias e Tecnológicas (FCAT), Sao Paulo State University (UNESP), Câmpus de Dracena, 17900-000 Dracena, SP, Brazil; ^2^Escuela Técnica Superior de Ingenieros Agrónomos, Universidad de Castilla La-Mancha, Campus Universitario, 02071 Albacete, Spain; ^3^Centro de Investigación, Experimentación y Servicios del Champiñón (CIES), Quintanar del Rey, 16220 Cuenca, Spain

## Abstract

*Agaricus subrufescens* Peck is a basidiomycete with immunomodulatory compounds and antitumor activities. This research evaluated the mycochemical composition of* A. subrufescens*, considering their morphological and physiological stage of maturity, with a particular focus on the development of a traceability process for the formulation of new nutritional products based on fungal foods. The stipes contained a high amount of dry matter (10.33%), total carbohydrate (69.56%), available carbohydrate (63.89%), and energy value (363.97 kcal 100 g^−1^ DM). The pilei contained a high amount of moisture (90.66%), nitrogen (7.75%), protein (33.96%), ash (8.24), crude fat (2.44%), acid detergent fiber (16.75 g kg^−1^), neutral detergent fiber (41.82 g kg^−1^), hemicellulose (25.07 g kg^−1^), and lignin (9.77 g kg^−1^). Stipes with mature physiological stage had higher values of dry matter (10.50%), crude fiber (5.94%), total carbohydrate (72.82%), AC (66.88%), and energy value (364.91 kcal 100 g^−1^ DM). Pilei of the mushrooms in the immature physiological stage had higher values of P (36.83%), N (8.41%), and A (8.44%). Due to the differences between the mycochemical compositions of the morphological parts of mushrooms linked to their physiological stage of maturity, such characteristics have immense potential to be considered for a traceability process. This study can be used for the purpose of providing the consumer with more product diversity, optimizing bioactivities of composts, and allowing farmers an efficient and profitable use of the mushroom biomass.

## 1. Introduction

*Agaricus subrufescens* Peck, which is synonymous with* Agaricus brasiliensis *(Wasser et al.) and* Agaricus blazei *(Murrill)* sensu* Heinemann [1], is a basidiomycete fungus commonly referred to as “almond mushroom,” “medicinal mushroom,” or “sun mushroom.” This mushroom has gained interest in the international scientific community because of its immunomodulatory compounds and antitumor activities [2–4].

Particular attention is required in the process of mushrooms cultivation designated for medicinal purposes [origin of the spawn (quality of the inoculum for production), compost and casing used, and production environment] accompanied with a detailed knowledge of mycochemical characteristics, based on morphological and physiological development of mushrooms, to improve their nutritional and pharmacological effect in humans consumption.

It has been shown that productivity of* A. subrufescens* is related to the quality of the compost, among others factors [5].

According to Wisitrassameewong et al. [6], various studies have attempted to optimize the composition of compost for mushroom cultivation. However, the association between the compost medium and nutritional quality of the harvested mushrooms has not been fully investigated. In this regard, Zied et al. [7] presented a detailed study on which process of mushroom cultivation influenced the amount of *β*-glucan in the mushrooms. The study aimed to create a cultivation protocol to standardize mushroom; however, the morphological and physiological development of the mushrooms were not emphasized in this research. These mushroom cultivation processes are important because they allow the grower to adopt practices that enhance the medicinal characteristics of the fungus.

**Figure 1:**
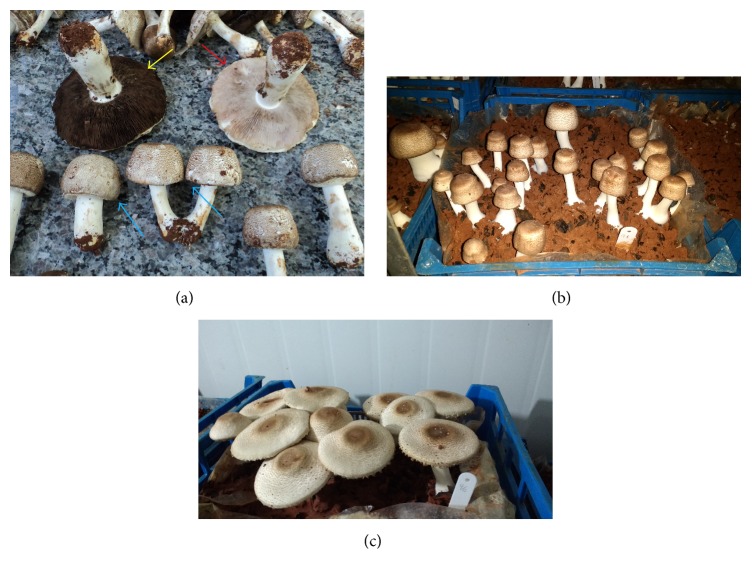
(a) Mushroom in different physiological stages (yellow arrow refers to mature mushroom with mature spore “dark brown spores”; red arrow refers to mature mushroom with immature spores; and blue arrow refers to immature mushrooms with closed pilei without lamella break). (b and c) Immature and mature mushrooms with immature spores used in the manuscript.

Morphologically, a mushroom can be divided into two main parts: pilei (superior part of the fruit body where the lamella and spores are located) and stipe (inferior part of the fruit body, consisting of a bulk sterile hyphal tissue) [8, 9]. Physiologically,* A. subrufescens* can be classified according to the following stages of maturity: immature (pilei closed), mature (pilei opened) with immature spores, and mature (pilei widely opened) with mature spores (Figure 1(a)) [10, 11].

We suggest that a systematic program of development could be devised, considering the morphological and physiological characteristics of mushrooms in traceability process. The definition of traceability process varies according to the industry sector, the current legislation, the country concerned, and the objectives of the process. For the agro-based food chain, food traceability can be defined as the information necessary to describe the production history of a food crop and any subsequent transformations or processes that the crop might be subject to in its distribution process from the grower to the consumer's plate, with detailed explanations of each stage [12, 13].

A targeted and more rigorous definition of food supply chain not specific to food commodities was provided by the International Organization for Standardization in 1994 [14] and supported by EC regulation 178/2002 [15]. This defines “traceability as the ability to trace and follow a food, feed, food producing animal or ingredients, through all stages of production and distribution.”

The production system and traceability of* A. subrufescens* cultivation involve three steps: the process of mushroom cultivation (studied by Zied et al. [7]), the morphological and physiological development of the mushroom (intended aim of this manuscript), and the postharvest method used for mushroom commercialization (dehydration, freeze-drying, extraction process of metabolic fractions, or bioactive compounds, etc.), a subject for future research.

Therefore, this study aimed to evaluate the mycochemical composition of* A. subrufescens*, considering their morphological and physiological development, with a particular focus on the development of a traceability process for the formulation of new nutritional products based on fungal foods.

## 2. Materials and Methods

The traceability system for mushrooms in Europe (Spain) was evaluated. The same strain, casing layer, and cultivation environment (chamber with controlled climate) were used, varying only the compost at each mushroom farm.

### 2.1. Traceability Process of Mushroom Cultivation

The* A. subrufescens *strain, ABL 99/30, was collected in 1999, from Piedade city, São Paulo State, Brazil, as described by Zied et al. [7]. Spawn was prepared according to the following steps: selection of mushroom, production of subculture, production of mother spawn, and production of grain spawn [16].

The* Agaricus bisporus* growing substrates, based on wheat straw and chicken manure, were provided by Compost Manchego (CM) SL (Villanueva de la Jara, Spain) and Compost Villacasa (CV) SL (Casasimarro, Spain).  Table 1 presents the analytical characteristics of the two substrates used, at the end of phase II of composting (pasteurization and aerobic thermophilic conditioning procedure). For each characteristic, four repetitions were performed.

After phase II of the composting process, the substrate was inoculated with* A. subrufescens* at 12 g kg^−1^ fresh compost. Euroveen® (BVB Substrates, Grubbenvorst, Netherlands), a Dutch commercial mixture, was used as casing material (Table 2). A 4 cm thick casing layer was applied 20 days after spawning.

Analyses of substrates and casing were carried out with four repetitions for each parameter in the Laboratory of the Centro de Investigación, Experimentación y Servicios del Champiñón (Quintanar del Rey, Spain). Competitor molds, mites, and nematodes were absent in both the composts and the Dutch commercial casing layer.

The crop cycle was conducted in an experimental chamber of the Centro de Investigación, Experimentación y Servicios del Champiñón (Quintanar del Rey, Spain), equipped with humidification systems, heating/cooling, and recirculating/outside air ventilation, which allowed automatic control of temperature, relative humidity (RH), and carbon dioxide concentration. After spawning, the compost was incubated at 28 ± 1°C, 95 ± 2% RH for 20 d, without external ventilation or lighting.

Ruffling was done eight days after casing application when the mycelia appeared on the surface. A day later, the environmental temperature (19 ± 1°C), RH (88 ± 2%), and carbon dioxide level (<800 ppm) were decreased, with illumination (150 lux, 12 h per d) provided to induce fruiting. After three days, the environmental temperature was increased to 24 ± 1°C until first flush cessation. To induce the second and third flush, the environmental temperature (19 ± 1°C) was decreased again for 3 d. The total duration of the crop cycle was 82 d.

### 2.2. Morphological and Physiological Development of Mushroom

Mushrooms, in two different stages of maturity (immature and mature) in the second flush, were harvested manually, totalizing 50 mushrooms in each stage. The immature mushrooms had the pilei with approximately 30 to 55 mm of diameter and the mature mushroom had the pilei with approximately 60 to 100 mm of diameter (Figures 1(b) and 1(c)). The mature mushrooms used were with immature spores. The bottoms of the stipes were scraped to remove casing layer residues. The immature and mature mushrooms were divided into three groups, according to morphological parts (pilei, stipes, and whole mushrooms); thus, a total of six parameters (pilei immature, stipes immature, whole mushrooms immature, pilei mature, stipes mature, and whole mushrooms mature) were analyzed, according to their mycochemical composition. Samples were dried at 50°C for 48 h and triturated for analysis. For each characteristic, six repetitions were performed.

### 2.3. Analyses in the Mushrooms, Composts, and Casing

Physical, chemical, and biological characteristics of mushrooms, composts, and casing layer were determined according to Pardo-Giménez et al. [17, 18]. The following measurements were taken:

- Moisture and dry matter contents (gravimetric method, drying at 103–105°C to a constant weight)
- pH (potentiometric method, extracting a test portion of sample with water at 22 ± 3°C; extraction ratios of 1 + 5 (W/V) and 1 + 5 (V/V) were used)
- Electrical conductivity (conductimetric method; a test portion is extracted with water at 22 ± 3°C in an extraction ratio of 1 + 5 (V/V) to dissolve the electrolytes)
- Total N content (Kjeldahl method, based on digestion with sulphuric acid/potassium sulphate)
- Protein (calculation by multiplying the total nitrogen content, obtained by the Kjeldahl method by a conversion factor of 6.25 for substrates and 4.38 for mushrooms)
- Organic matter and ash (thermogravimetric method, measuring the loss of weight after calcination at 540°C)
- C/N ratio (calculation from organic matter and nitrogen contents; the conversion of organic matter into its carbon content is made based on the assumption that organic matter contains 58% organic carbon)
- Crude fiber (Weende technique adapted to the Ankom filter bag technique; this method determines the organic residue remaining after digestion with solutions of sulphuric acid and sodium hydroxide)
- Crude fat (gravimetric method adapted to the Ankom filter bag technique, extracting the sample with petroleum ether)
- Total carbohydrates (calculation by subtracting the sum of the crude protein, total fat, water, and ash from the total weight of the material)
- Nitrogen-free extracts/available carbohydrate content (calculation by subtracting the crude fiber from the total carbohydrate content)
- Energy value (calculation from the relative content of protein, fat, and carbohydrates using modified Atwater factors)
- Cellulose, hemicellulose, lignin, and acid and neutral detergent fiber (Van Soest fiber detergent methods: acid and neutral detergent fiber method by Ankom filter bag technique and Ankom acid detergent lignin in beakers method were used)
- Particle real density (calculation from ash content considering that the density of the organic matter is 1.55 kg m^−3^ and that of the ashes 2.65 kg m^−3^)
- Bulk density, fresh (weight of certain volume of material after compaction, determined in laboratory using a cylinder of 1L of capacity by compacting the sample under defined conditions)
- Bulk density, dry (calculation from fresh bulk density and moisture content)
- Total of pore space (calculation from dry bulk density and real density)
- Water holding capacity (saturation and drainage method, determined from the moisture content of the sample after being subjected to three cycles of saturation and drainage at atmospheric condition)
- Active lime (ammonium oxalate method, calculated by means of a gasometric dosage of the CO_2_ of the ammonium carbonate, formed by reacting the calcium carbonate of the sample with a solution of ammonium oxalate with the aid of a Bernard calcimeter)
- Total carbonates (hydrochloric acid method, based on the measurement of the volume of CO_2_ released when treating the carbonates with hydrochloric acid)

### 2.4. Statistical Analysis

ANOVA was used to analyze the data, and the Fisher's LSD test was employed to establish significant differences between means (*p* ≤ 0.05). All calculations were performed using the Statgraphics Plus software, v. 4.1 (Statistical Graphics Corp., Princeton, NJ, USA). PCA (Principal Component Analysis) was performed with R software [19], to reduce the dimensionality of the data and to identify components which allow discriminating the material.

## 3. Results and Discussion

Table 3 shows the ANOVA results for the 16 mycochemical characteristics, considering the type of compost (a), morphological mushroom parts (b), and physiological stage of mushroom maturity (c).  Table 4 shows the respective mean tests for the variables that were significant by ANOVA, to describe the traceability process linking the cultivation information (using the two composts) and its response to the harvested mushrooms composition.

The compost is an important factor in the quality (physical, chemical, and biological) of mushroom [17]. However, in this study, a comparison of the two composts (CM and CV) indicated significance for only five mushroom characteristics: CFa (crude fat), EV (energy value), ADF (acid detergent fiber), CE (cellulose), and LI (lignin), as shown in Table 3(a). The reasons that only those characteristics showed differences are unclear, particularly regarding the percentage of CFa and CE, that composts with a higher percentage of these elements result in harvested mushrooms with lower value of these elements, which makes us understand that there is no direct correlation between CFa and CE content in the compost and harvested mushroom.

It should be noted that the characteristics analyzed in the substrates do not differ drastically (Table 1), the composts being quite similar, meeting the compost quality index for mushroom cultivation [20]. Pardo et al. [21] introduce a HACCP system in the compost elaboration line, which allow the composting companies to design and establish a self-control system to ensure the quality of their productions, but the authors did not verify the mycochemical characteristics of the harvested mushrooms. Zied and Minhoni [22] obtained the highest protein content in mushrooms cultivated on substrate with high protein content, a result not verified in the present manuscript.

Food traceability systems are therefore becoming critical for the food industry and the public sector, as well as for consumers. The increased requirements for documentation and reporting systems are taking a toll on developing countries that are hoping to expand their trade in food or break into new markets [23].

Concerning the morphological characteristics of the mushrooms (stipes, pilei, and whole mushrooms), the results (Table 3(b)) indicated that only CFi (crude fiber) and CE were not significantly different from each other, by the *F* test (*p* < 0.05). Regarding the physiological characteristics, all variables were significantly different, depending on the stage of mushroom maturation (Table 3(c)). The means test results of the characteristics that were significant by ANOVA are presented in Table 4, associated, respectively, with the morphological parts of the mushroom (b) and physiological stage of maturity (c). The results show the importance of this study in characterizing the distribution of mycochemicals within the mushroom.

Notably, the stipes contained a high amount of dry matter (DM), total carbohydrate (TC) and available carbohydrate (AC), and energy value (EV). Furthermore, the pilei had a high amount of moisture (M), nitrogen (N), protein (P), ash (A), CFa, ADF, neutral detergent liquid (NDF), hemicellulose (HE), and LI (Table 4(b)). These results highlight the wide variation of particular characteristics based on the morphological parts of the mushroom, such as N (36%), P (36%), CFi (29%), ADF (33%), NDF (35%), HE (36%), and LI (55%).

In contrast to the results verified in this study, Mol and Wessels [24] studied the differences in wall structure between substrate hyphae and hyphae of fruit-body stipes in* Agaricus bisporus* and concluded that there was no significant difference in gross chemical wall composition of the two hyphal types. However, Jasinghe and Perera [25] found a varying amount of ergosterol depending on the morphological part of shiitake mushrooms, accumulating more in the gills than in the stipe.

According to the review of Manning [26], on the chemical composition and nutritional value of cultivated mushrooms, carbohydrates are the main component of mushrooms apart from water and account for an average of 4.2% of the fresh weight. Glycogen and HE are the main polysaccharides found in mushrooms; contents of 8.18% (dry weight) of HE have been recorded in* A. campestris* [26], markedly lower than those obtained in this research with* A. subrufescens* (between 14.58 and 26.22%) (Table 4(c)). Pardo et al. [27] studied different strains and composts of* A. subrufescens *and found HE values between 17.5 and 22.7%.

To better understand the composition of the mushrooms, we also examined their physiological characteristics to provide a complete mycochemical profile of* A. subrufescens *(Table 4(c)). We observed that the stipes of the mushrooms with opened pilei (mature stipes) had higher values of DM, CFi, TC, and AC compared to equivalent stipes of the mushrooms with closed pilei (immature stipes). The values increased in proportion to the physiological fungus maturity.

Pilei of the mushrooms in the immature physiological stage had higher values of DM, P, N, and A compared with equivalent pilei in mushrooms at the mature physiological stage. The values decreased in proportion to the physiological fungus maturity.

The results of the whole mushrooms represent intermediate values between the stipes and pilei for all mycochemical characteristics analyzed. The physiological characteristics were more influenced than the morphological characteristics, according to the maturity of the mushroom, with the following values obtained: N (49%), P (49%), CFa (49%), AC (29%), NDF (42%), ADF (42%), HE (44%), and LI (64%).

A limited number of studies have reported the tissue distribution of bioactive metabolites in mushrooms during development, despite increasing evidence that maturation affects the concentration of natural compounds in mushrooms [28]. Camelini et al. [10] emphasized that the yield and structural diversity of glucans in* Agaricus brasiliensis* increased in the fruiting bodies matured. The amount of (1→3)-*β*-glucans in the mature stage was higher than in the immature stage [10]. According to Chang and Miles [29], the amount of (1→4)-*β*-glucan in the mushroom can also be associated with the amount of cellulose, with the highest amount being found in the stipes of immature mushrooms (7.29%) and in the immature of whole mushroom (7.21%), which may justify the market of this mushroom with closed pilei.

**Figure 2:**
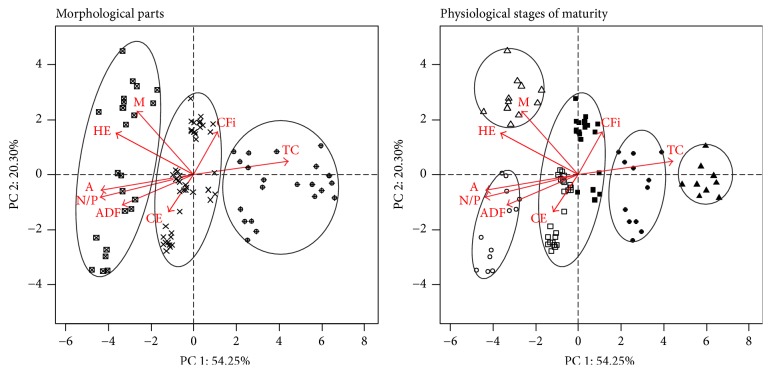
Score plot obtained from PCA that allowed the distinction of the evaluated material with respect to its morphological parts, *⊠*: pilei; ×: whole mushrooms; ⊕: stipes, and with respect to its physiological stages of maturity, ∘: immature pilei; •: immature stipes; △: mature pilei; ▲: mature stipes; □: immature whole mushrooms; ■: mature whole mushrooms. In the second scatter plot, the evaluated characteristics do not allow the complete distinction between samples of immature and mature whole mushrooms.

As this study aimed to develop a traceability process for* A. subrufescens* and assuming that the variables evaluated were necessary and sufficient, a PCA was performed to obtain components to discriminate the evaluated material. The first and the second estimated principal components (PC1 and PC2) retained 74.56% of the variance contained in the original variables (Figure 2). It is important to note that the dispersion is not due to a specific characteristic, but that the characteristics are combined, requiring full evaluation to attain traceability of the material.

In estimating the principal components, the variables with the highest weighting in the smallest eigenvalue are considered of minor importance in explaining the variability of the studied material. Thus, minor variables, in decreasing order, are AC, NDF, HE, and EV. The scatter plot analysis of the first two principal components showed three major regions (Figure 2, morphological parts), associated with the morphological parts of mushrooms: the left region, which had the lowest value for PC1, was associated with the pilei of the mushrooms; the opposite region, which had the highest value for PC1, was associated with the stipes; and the central region was associated with the whole mushrooms.

Figure 2 (physiological state of maturity) shows a subdivision of the evaluated characteristics with regard to the physiological stage of mushroom maturity. In this instance, there was a subdivision of the materials related to the morphological parts, stipes, and pilei, respectively. The pilei could be separated, by means of PC2, into mature (positive values) and immature (negative values). For the region associated with the stipes, there was also a subdivision but, in this instance, it was associated with PC1, in which highest values were correlated to mature stipes.

From interpretation of the scatter plot, the results suggested that the pilei had a higher content of EV, M, HE, A, N, P, and ADF, compared to the stipes and whole mushrooms. Still, higher EV, M, and HE values were present when the pilei were opened and higher amounts of A, P, N, and ADF were present when the pilei were closed. CFi and CE values were typically associated with the whole mushrooms, with a slight slope of CFi in the opened mushrooms and CE in the closed pilei (understood in this situation that the closed pilei had a greater amount of *β*-glucan). The highest amount of TC was found in the stipes with pilei opened.

Previous literature has shown that the antioxidant activity was highest during early developmental stages in button mushrooms,* Agaricus bisporus* [30], but no explanation was provided. In our study, we propose that the concentration of some components in specific morphological parts (i.e., pilei) of the mushroom is related to biochemical processes involved in the reproduction of fungi, mainly formation and maturation of the spores (events of karyogamy, meiosis, and sporogenesis). Karyogamy is a fusion of two haploid nuclei. Meiosis is a reduction division where chromosome number in the two nuclei is reduced to half and variability is generated due to random distribution of chromosomes from the parents. Sporogenesis is like mitotic division in which chromatids separate and variability generated through crossing over of chromatids at chromosome pairing stage is released. As a result, each of the four nuclei produced after meiosis contains different combination of alleles. It would be particularly important to conduct a detailed study focused on the mycochemical characteristics in pilei, separating the gills from the remainder of the pileus, to improve the traceability process.

The traceability process presented in this manuscript allowed the identification of differences between morphological and physiological characteristics of mushroom with respect to their mycochemical compositions and distribution. This methodology is extremely important for the formulation of new nutritional products to be applied in specific diets based on fungal foods, such as diets with high protein and mineral “ash” content (use of pilei) or diets with high carbohydrates and energy value content (use of stipes). However, more research is needed in these topics of study.

## 4. Conclusions

Due to the differences between the mycochemical compositions of the morphological parts of mushrooms linked to their physiological stage of maturity and the type of compost (with minor significance), such characteristics have immense potential to be considered for a traceability process. This study can be used for the purpose of providing the consumer with more diversity and news products, optimizing bioactivities of composts, and allowing farmers an efficient and profitable use of the mushroom biomass.

## Figures and Tables

**Table 1:** Analytical characteristics of the substrates at the end of phase II. CM corresponds to Compost Manchego and CV to Compost Villacasa.

Parameter	CM	CV
pH (1 : 5, w/v)	7.63 ± 0.02	7.44 ± 0.02
Moisture (g kg^−1^)	664.7 ± 3.8	654 ± 3.3
Total nitrogen (g kg^−1^ d.m.)	19.7 ± 0.9	21.3 ± 0.7
Protein (g kg^−1^ d.m.)	123.1 ± 5.4	132.9 ± 4.1
Ash (g kg^−1^ d.m.)	237.2 ± 2.4	224.6 ± 6.5
Organic matter (g kg^−1^ d.m.)	762.8 ± 2.4	775.4 ± 6.5
Carbone/Nitrogen	22.5 ± 1.0	21.2 ± 0.6
Crude fiber (g kg^−1^ d.m.)	330 ± 17.8	292.8 ± 9.6
Crude fat (g kg^−1^ d.m.)	2.5 ± 0.8	3.3 ± 0.7
N-free extracts (g kg^−1^ d.m.)	307.2 ± 15.5	346.4 ± 10.3
Total carbohydrates (g kg^−1^ d.m.)	637.2 ± 6.7	639.2 ± 7.1
Hemicellulose (g kg^−1^ d.m.)	123.4 ± 1.3	106.8 ± 5.7
Cellulose (g kg^−1^ d.m.)	204.8 ± 15.8	190.3 ± 5.7
Lignin (g kg^−1^ d.m.)	208.8 ± 11.1	226.7 ± 3.8
Neutral-detergent sol. fiber (g kg^−1^ d.m.)	225.8 ± 12.4	251.6 ± 13.0

Each value is expressed as mean ± standard deviation (*n* = 4); d.m.: dry matter.

**Table 2:** Physical and chemical characteristics of Dutch commercial casing.

Parameter	Value
pH (1 : 5, v/v)	8.26 ± 0.05
Electrical conductivity (1 : 5, v/v; *μ*S cm^−1^)	231 ± 2
Particle real density (g mL^−1^)	1.759 ± 0.009
Ash (g kg^−1^)	286.7 ± 9.4
Bulk density, wet (g mL^−1^)	0.613 ± 0.009
Moisture (g kg^−1^)	831 ± 0.8
Bulk density, dry (g mL^−1^)	0.104 ± 0.001
Total pore space (mL L^−1^)	941 ± 1
Water-holding capacity (kg kg^−1^)	6.97 ± 0.31
Organic matter (g kg^−1^)	713.3 ± 9.4
Nitrogen (g kg^−1^)	8.6 ± 0.3
Carbone/nitrogen	48.1 ± 0.8
Total carbonates (g kg^−1^)	137 ± 7
Active lime (g kg^−1^)	55 ± 2

Each value is expressed as mean ± standard deviation (*n* = 4).

**(a):** 

		Mean Square^1^														
	df	DM	M	N	P	A	CFi	CFa	TC	AC	EV	ADF	NDF	HE	CE	LI
Treatments	1	0.27^ns^	0.34^ns^	0.02^ns^	0.32^ns^	0.39^ns^	0.09^ns^	4.86^*∗∗∗*^	1.07^ns^	1.74^ns^	223.05^*∗∗∗*^	111.84^*∗∗*^	10.63^ns^	53.46^ns^	1.39^*∗*^	94.29^*∗∗*^

Mean		9.76	90.13	6.49	28.44	7.67	5.60	2.13	61.65	56.05	359.55	14.22	35.30	21.08	7.00	7.33
CV (%)		5.73	0.65	18.18	18.19	6.98	6.64	25.34	9.30	10.01	1.20	22.14	16.49	18.39	7.88	41.58

**(b):** 

		Mean Square^1^														
	df	DM	M	N	P	A	CFi	CFa	TC	AC	EV	ADF	NDF	HE	CE	LI
Treatments	2	6.16^*∗∗∗*^	5.94^*∗∗∗*^	47.66^*∗∗∗*^	914.30^*∗∗∗*^	9.55^*∗∗∗*^	0.08^ns^	3.24^*∗∗∗*^	1241.90^*∗∗∗*^	1222.10^*∗∗∗*^	355.40^*∗∗∗*^	193.04^*∗∗∗*^	1301.40^*∗∗∗*^	491.90^*∗∗∗*^	0.15^ns^	180.11^*∗∗∗*^

Mean		9.76	90.13	6.49	28.44	7.67	5.60	2.13	61.65	56.05	359.55	14.22	35.30	21.08	7.00	7.33
CV (%)		4.43	0.53	9.54	9.55	3.87	6.66	24.71	4.15	4.22	1.03	18.70	7.15	10.81	8.07	34.84

**(c):** 

		Mean Square^1^														
	df	DM	M	N	P	A	CFi	CFa	TC	AC	EV	ADF	NDF	HE	CE	LI
Treatments	5	4.96^*∗∗∗*^	3.98^*∗∗∗*^	25.91^*∗∗∗*^	497.10^*∗∗∗*^	4.87^*∗∗∗*^	0.85^*∗∗∗*^	4.98^*∗∗∗*^	612.10^*∗∗∗*^	585.60^*∗∗∗*^	265.42^*∗∗∗*^	87.12^*∗∗∗*^	553.60^*∗∗∗*^	216.21^*∗∗∗*^	1.17^*∗∗*^	76.62^*∗∗∗*^

Mean		9.76	90.13	6.49	28.44	7.67	5.60	2.13	61.65	56.05	359.55	14.22	35.30	21.08	7.00	7.33
CV (%)		2.40	0.42	2.01	2.01	2.37	5.59	13.44	0.99	1.13	0.75	18.28	6.17	9.82	7.40	34.73

^1^Significance codes: ^*∗∗∗*^*p* < 0.001; ^*∗∗*^*p* < 0.01; ^*∗*^*p* < 0.05; ^ns^*p* > 0.05. CV: coefficient of variation; df: degree of freedom.

**(a):** 

	DM	M	N	P	A	CFi	CFa	TC	AC	EV	ADF	NDF	HE	CE	LI
MC	—	—	—	—	—	—	2.35^a^	—	—	361.07^a^	13.14^b^	—	—	6.88^b^	6.34^b^
VC	—	—	—	—	—	—	1.90^b^	—	—	359.02^b^	15.30^a^	—	—	7.12^a^	8.31^a^

*Note*. MC: Manchego Compost; VC: Villacasa Compost.

**(b):** 

	DM	M	N	P	A	CFi	CFa	TC	AC	EV	ADF	NDF	HE	CE	LI
Stipes	10.33^a^	89.67^c^	4.96^c^	21.73^c^	6.99^c^	5.67	1.72^b^	69.56^a^	63.89^a^	363.97^a^	11.13^c^	27.25^c^	16.12^c^	—	4.34^c^
Wh. Mush.	9.69^b^	90.09^b^	6.63^b^	29.03^b^	7.73^b^	5.58	2.18^a^	60.83^b^	55.25^b^	358.83^b^	14.50^b^	36.07^b^	21.57^b^	—	7.60^b^
Pilei	9.34^c^	90.66^a^	7.75^a^	33.96^a^	8.24^a^	5.56	2.44^a^	55.36^c^	49.80^c^	356.54^c^	16.75^a^	41.82^a^	25.07^a^	—	9.77^a^

*Note.* Wh. Mush: whole mushroom.

**(c):** 

	DM	M	N	P	A	CFi	CFa	TC	AC	EV	ADF	NDF	HE	CE	LI
Mat. Stipes	10.50^a^	89.50^c^	4.32^e^	18.91^e^	6.68^e^	5.94^a^	1.59^c^	72.82^a^	66.88^a^	364.91^a^	10.10^d^	24.69^d^	14.58^e^	6.54^b^	3.71^d^
Imm. Stipes	10.17^b^	89.83^bc^	5.61^d^	24.55^d^	7.29^d^	5.41^bc^	1.85^c^	66.31^b^	60.90^b^	363.03^ab^	12.16^cd^	29.82^c^	17.66^d^	7.29^a^	4.98^cd^
Mat. Pilei	8.81^e^	91.19^a^	7.10^b^	31.10^b^	8.04^b^	5.73^ab^	3.12^a^	57.75^e^	52.02^e^	360.82^b^	16.13^ab^	42.35^a^	26.22^a^	6.99^ab^	9.22^ab^
Imm. Pilei	9.88^c^	90.12^b^	8.41^a^	36.83^a^	8.44^a^	5.39^c^	1.76^c^	52.97^f^	47.58^f^	352.27^d^	17.37^a^	41.29^a^	23.92^ab^	7.15^ab^	10.32^a^
Mat. Wh. Mush.	9.37^d^	90.18^b^	6.09^c^	26.67^c^	7.52^c^	5.78^a^	2.56^b^	62.80^c^	57.02^c^	360.64^b^	13.94^bc^	35.94^b^	22.00^bc^	6.80^ab^	7.25^bc^
Imm. Wh. Mush.	10.01^bc^	89.99^b^	7.17^b^	31.40^b^	7.93^b^	5.40^c^	1.80^c^	58.87^d^	53.47^d^	357.03^c^	15.06^ab^	36.21^b^	21.15^c^	7.21^a^	7.95^ab^

*Note.* Mat: mature; Imm.: immature; Wh. Mush: whole mushroom.
